# Case Report: Predominant Tubulointerstitial Lupus Nephritis or the Combination With IgG4-Related Disease?

**DOI:** 10.3389/fmed.2021.684889

**Published:** 2021-06-28

**Authors:** Ying Tan, Yan Qin, Xiao-juan Yu, Rong Xu, Su-xia Wang, Fu-de Zhou, Ming-hui Zhao

**Affiliations:** ^1^Renal Division, Department of Medicine, Peking University First Hospital, Beijing, China; ^2^Institute of Nephrology, Peking University, Beijing, China; ^3^Key Laboratory of Renal Disease, Ministry of Health of China, Beijing, China; ^4^Key Laboratory of CKD Prevention and Treatment, Ministry of Education of China, Beijing, China; ^5^Research Units of Diagnosis and Treatment of Immune-mediated Kidney Diseases, Chinese Academy of Medical Sciences, Beijing, China; ^6^The Second People's Hospital of Shanxi Province, Shanxi, China; ^7^Laboratory of Electron Microscopy, Pathological Center, Peking University First Hospital, Beijing, China; ^8^Peking-Tsinghua Center for Life Sciences, Beijing, China

**Keywords:** systemic lupus erythematosus, lupus nephritis, predominant interstitial nephritis, IgG3, storiform pattern

## Abstract

Isolated or dominant tubulointerstitial lupus nephritis is rare. Here, we reported a 67-year-old man diagnosed with systemic lupus erythematosus (SLE) based on clinical and laboratory criteria, who was showing impaired renal function and non-nephrotic range proteinuria in the past 2 years. Renal biopsy showed almost normal glomeruli, but the tubulointerstitium showed “storiform” pattern with interstitial infiltration of IgG3 predominant plasma cells. Immunofluorescence showed linear and granular staining of IgG and C1q along TBM and interstitium. He started on medium dose of oral steroids and mycophenolate mofetil, which were gradually tapered. As a result, his renal function improved over a few days. Now, he continued on low dose steroids and mycophenolate mofetil with no evidence of relapse.

## Introduction

Systemic lupus erythematosus (SLE) is a multisystemic immunological disorder with various manifestations. Lupus nephritis is the most common manifestation of SLE and contributes significantly toward morbidity and mortality in this disease. Tubulointerstitial involvement has been observed in ~90% of all patients with lupus nephritis^1^. However, the predominant or isolated presence of tubulointerstitial changes in the setting of minimal glomerular abnormalities in patients with SLE is rare. Here, we describe a case of predominant tubulointerstitial lupus nephritis, which was diagnosed with SLE based on clinical and laboratory criteria. The patient presented with impaired renal function, sub-nephrotic proteinuria, and evidence of tubular dysfunction.

## Case Report

In 2018, a 67-year-old Chinese man was admitted to our hospital with dryness of mouth for 6 years and elevated serum creatinine for 2 years. Six years ago, he felt dryness of mouth. Enlargement of salivary gland were discovered, and resection of bilateral submandibular gland was performed. The pathologic examination revealed severe chronic inflammation. Two years ago, the patient felt fatigue. The serum creatinine was 164 μmol/L (corresponding to estimated glomerular filtration rate of 40.2 ml/min/1.73 m^2^ as calculated by the CKD-EPI equation) with benign urinary analysis. Half a year later, his serum creatinine elevated to 175 μmol/L (corresponding to estimated glomerular filtration rate of 37.5 ml/min/1.73 m^2^ as calculated by the CKD-EPI equation). Positive antinuclear antibody (ANA) with titer of 1:3,200 was discovered. Oral methyprednisolone was prescribed as 32 mg per day and tapered quickly because of deteriorating dry mouth. After that, his serum creatinine suddenly elevated to 517 μmol/L within 3 months. And he had intermittent facial rash when exposed to the sun.

The patient had tuberculous pleurisy 50 years ago and fully recovered. Family history was of no significance.

On admission, physical examination revealed blood pressure of 125/70 mmHg, temperature of 36.3°C, pulse of 75 beats/min, and respiratory rate of 15/min. His tongue was dry, and dental caries could be found.

Laboratory data were as follows. Urinary analysis showed proteinuria+, glucose+ with normal blood glucose. Urine sediment analysis revealed red blood cell 0–2 cells per high power field (HPF) with white blood cell 0–1/HPF. Urinary protein excretion was 1.00 g/24 h (urine volume 2,700 ml). The urinary alpha 1 microglobulin was 343 mg/L (normal range: 0.00–12.00 mg/L) and N-Acetyl-D-Glucosaminidase 22 IU/L (normal range: 0–21 IU/L). Urine osmotic pressure was 314 mOsm/kg with normal blood osmotic pressure. Urine protein electrophoresis showed 58.90% of small molecule protein, 32.9% of albumin, and 8.20% of large molecule protein. Blood routine test showed mild anemia (hemoglobin 95 g/L). Blood chemistry tests showed increased levels of blood urea nitrogen and serum creatinine (16.91 mmol/L and 456.00 μmol/L, respectively). Serological studies revealed a high titer of ANA (1:10,000) and an increased level of anti-dsDNA antibody (>800 IU/ml) with negative ENA. Decreased serum levels of C3 (0.240 g/L) and C4 (<0.017 g/L) were detected. Total IgG was elevated to 33.80 g/L, and IgG subtypes were IgG1 19.8 g/L, IgG2 3.84 g/L, IgG3 2.24 g/L, and IgG4 2.43 g/L. Coombs test was positive.

Tear break-up time (BUT) indicated dryness of eye (<10 s). Labial gland biopsy did not indicate the diagnosis of Sjogren's Syndrome. Submandibular gland pathology revealed severe chronic inflammation of the left submandibular gland. Immunochemistry staining of immunoglobulin showed IgG3 positive.

**Figure 1:**
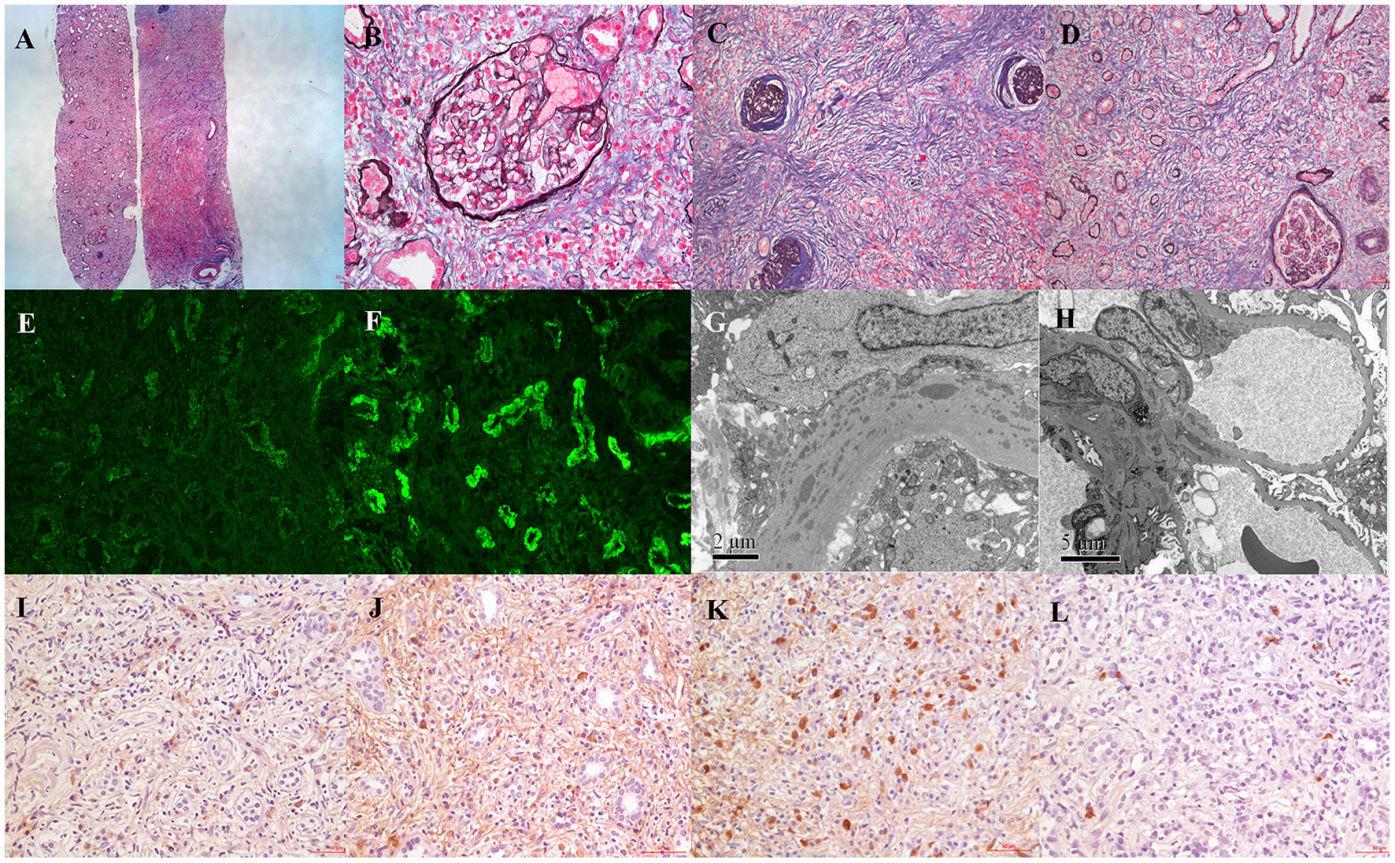
Patient renal biopsy findings. **(A)** Light microscopy showed minor shrinkage of glomerular capillary wall with dense interstitial lymphoplasmacytic cell infiltrates and variable degree of interstitial fibrosis (periodic methenamine silver and Masson trichrome staining, ×40). **(B)** Almost normal glomeruli (periodic methenamine silver and Masson trichrome staining, ×400). **(C,D)** “Storiform” fibrosis with tubular atrophy in the tubulointerstitium (periodic methenamine silver and Masson trichrome staining, ×200). **(E)** Immunofluorescence staining showed IgG granular staining along TBM and interstitium (×400). **(F)** Immunofluorescence staining showed λ linear staining along TBM (×400). **(G)** Electronic dense deposits along tubular basement membrane (×12,000). **(H)** Electronic dense deposits in the mesangium (×12,000). **(I–L)** Immunohistochemical staining of IgG subclass (IgG1–IgG4) (×200).

Percutaneous renal biopsy was performed. Light microscopy examination showed that 3/14 glomeruli were globally sclerosed and 6/14 glomeruli showed ischemic sclerosis. Other glomeruli showed minor shrinkage of glomerular capillary wall, but no significant glomerular change. Tubular epithelial cells exhibited focal vacuolization and eosinophilic granules in the cytoplasm and focal loss of brush border. There were profound interstitial infiltration with lymphocytes, plasma cells, and a few eosinophils. “Storiform” fibrosis with tubular atrophy can be seen in the tubulointerstitium (Figures 1A–C). Immunofluorescence microscopy revealed one out of seven glomeruli with crescent and granular mesangial staining for IgG and C3, and granular staining for IgG, κ, λ, IgG1, IgG2, IgG3, IgG4, and C1q along TBM and interstitium and linear staining for λ also along TBM (Figure 1D). Electron microscopy revealed one glomerulus with numerous electronic dense deposits along tubular basement membrane with focal mesangial deposits (Figure 1E). Immunohistochemistry revealed that IgG1-positive plasma cells were 35–45/HPF, IgG2-positive plasma cells 40–50/HPF, IgG3-positive plasma cells 90–100/HPF, and IgG4-positive plasma cells 10–15/HP (Figures 1F–I). Taken together with the diagnosis of SLE, these findings indicated the renal lesion as predominant acute and chronic tubulointerstitial nephritis with dominant IgG3 positive plasma cell infiltration.

Oral prednisone 30 mg was prescribed immediately. Hydroxychloroquine 0.2 g and mycophenolate mofetil 1.0 g were given later. The serum creatinine was gradually reduced from 456 μmol/L to 210 μmol/L. The urinary protein was reduced to 0.17 g/24 h in 8 months. The patient was stable for the last 31 months during follow-up.

## Discussion and Literature Review

The 2003 International Society of Nephrology/Renal Pathology Society (ISN/RPS) system for classifying patients with lupus nephritis was based on glomerular lesions. However, previous studies have indicated that severe tubulointerstitial involvement has been observed in approximately more than half of all patients with lupus nephritis and helpful in predicting renal outcome in patients with lupus nephritis (1). Major or isolated tubulointerstitial damage is rare.

Here, we presented a patient with SLE who presented with acute onset of chronic disease attributable to predominant tubulointerstitial lupus nephritis. The diagnosis of lupus was based on the presence of 5 of the 11 criteria for the diagnosis of SLE (photosensitivity, autoimmune hemolytic anemia, a positive ANA, a positive anti-dsDNA antibody, and the presence of renal disease). In addition, the patient was noted to have hypocomplementemia and hyperglobulinemia.

The chief complaint of the patient was dryness of mouth. Taking the enlargement of salivary gland, we needed to exclude primary Sjögren's syndrome. The specific antibody of Sjögren's syndrome and anti-SSA and anti-SSB antibodies were negative. Though the dryness of eyes was diagnosed by BUT, the labial gland biopsy did not meet the criteria of primary Sjogren's Syndrome (2). Moreover, we also needed to exclude IgG4 disease since the elevated IgG4 (≥135 mg/dl), enlargement of salivary gland, and the kidney biopsy with storiform fibrosis and a lymphoplasmacytic infiltration. Thus, the immunohistochemistry of IgG subtype on tubulointerstitial plasma cells and submandibular gland was performed with all IgG3 predominant, and the ratio of IgG4/IgG <40%, which did not support IgG4-related disease (IgG4-RD) (3–5). However, there's still possibility that there might be an overlap of IgG4-RD and SLE since “storiform fibrosis” was more prevalent in IgG4-RD and more research work needs to be done to further differentiate the new disease entity (6, 7).

Meanwhile, a detailed history did not reveal the consumption of any potentially nephrotoxic medications or exposure to radiation or environmental toxin (e.g., cadmium or lead). Furthermore, though the renal biopsy revealed a few eosinophils in the interstitial infiltrate, it was not suggestive of an allergic interstitial nephritis. Similarly, there was no evidence of an infection, a neoplastic process, or a metabolic factor, which could have accounted for the current presentation.

Our patient was a predominant tubulointerstitial lupus nephritis with normal glomeruli. Fifteen similar previously reported cases are summarized in Table 1: acute kidney injury or tubular acidosis with trace proteinuria as onset. Renal pathology showed no or only slight glomerular lesions, but interstitial fibrosis, inflammatory cell infiltration, and renal tubular atrophy. Immunofluorescence showed IgG, C1q, and C3 deposition along TBM. Electron microscopy showed electron dense deposits on TBM. Most patients had a good prognosis with steroids just like our patient (8–21) (Table 1).

**Table 1:** Reported cases of predominant tubulointerstitial lupus nephritis.

**No. of case**	**Age (yrs)**	**Sex**	**Presentation**	**Urine analysis**	**LM**	**IF**	**EM**	**Therapy and prognosis**
1(4)^a^	52	F	AKI	2+ protein	Interstitial fibrosis and mononuclear cell infiltration; tubular atrophy; glomeruli appeared almost normal	Granular IgG, IgM, C3 in TBM; faint C3 in mesangium	EDD in TBM	Corticosteroid (improved)
2(5)^a^	23	F	Tubular acidosis	No proteinuria	Focal interstitial fibrosis and cell infiltration; tubular atrophy; normal glomeruli	C3 in TBM; negative in glomeruli	(-)	Not affected
3(6)^a^	30	F	AKI	No proteinuria	Interstitial fibrosis and mononuclear cell infiltration; tubular atrophy; mild mesangial cell proliferation	Granular IgG, C3 in TBM; interstitium, mesangium	EDD in mesangium	Corticosteroid (improved)
4(7)^a^	42	F	AKI	Mild proteinuria	Interstitial fibrosis and mononuclear cell infiltration; tubular atrophy; mild mesangial cell proliferation.	Granular IgG, Clq in TBM; small IgG, Clq, C3 in mesangium	(-)	Corticosteroid (improved)
5(7)^a^	24	F	AKI	Proteinuria	Interstitial fibrosis and mononuclear cell infiltration; ischemic glomeruli; mild mesangial cell proliferation	Granular IgG, Clq, C3, IgM in TBM; negative in glomeruli	(-)	Peritoneal dialysis (improved)
6(8)^a^	72	M	RD + NS	4+ protein	Interstitial fibrosis; minimal change in glomeruli	IgG, IgM, C3 in interstitium	Fusion of foot process	Corticosteroid and cyclophosphamide (death from sepsis)
7 (9)^a^	3	M	Without RD	1+ protein	Interstitial fibrosis and mononuclear cell infiltration; tubular atrophy; mild increase in mesangial cell and matrix	Linear IgG in TBM; granular IgG, C3, Clq in mesangium	EDD in mesangium, not in TBM	Corticosteroid (stable)
8 (10)^a^	25	F	AKI	1+ protein	Interstitial fibrosis and mononuclear cell infiltration; tubular atrophy; minimal change in glomeruli	C3 in TBM; mild C3 in mesangium	No EDD	Supportive therapy (stable)
9 (11)^a^	59	M	AKI	Mild proteinuria	Interstitial fibrosis and mixed cellular infiltration; tubular atrophy; normal glomeruli	Granular IgG, IgA, IgM, C3, Clq in TBM; negative in glomeruli	EDD in TBM	Corticosteroid (improved)
10 (12)^a^	30	F	RD	Mild proteinuria	Interstitial edematous and mononuclear cell infiltration; tubular atrophy; minimal change in most glomeruli	Granular IgG, C3, Clq in TBM	(-)	Corticosteroid (improved)
11 (13)^a^	64	M	RD	Mild proteinuria	Interstitial fibrosis and mononuclear cell infiltration; tubular atrophy; minimal change in glomeruli	Granular IgG, C3, Clq in TBM	EDD in TBM	Corticosteroid (improved)
12 (14)^a^	63	M	Without RD	No proteinuria	Interstitial fibrosis and mononuclear cell infiltration; tubular atrophy; mild increase in mesangial cell	Granular IgG, IgA, IgM, C3, Clq in TBM; interstitium, mesangium	EDD in mesangium and in TBM	Corticosteroid (stable)
13 (15) ^a^	67	F	AKI	Mild proteinuria	Focal tubular atrophy, abundant lymphocyte infiltrates, mostly from plasma cells. No significant glomerular alterations.	Granular deposits in the arterial walls of C3 and tubular cylinders of IgA	(-)	Corticosteroid (improved)
14 (16)^a^	38	M	RD	Mild proteinuria	Interstitial mononuclear cell infiltration; normal glomeruli	IgG, IgA, Clq, C3in TBM and PTCBM; negative in glomeruli	(-)	Corticosteroid (improved)
15 (17) ^a^	18	F	RD	Mild proteinuria	Diffuse and focally destructive, focally enhanced tubulointerstitial inflammatory infiltrate,Minimal glomerular changes with slight mesangial hypercellularity.		Mesangial deposits	Corticosteroid and cyclo phosphamide/MMF (improved)

*AKI, acute kidney injury; RD, renal deficiency; NS, nephrotic syndrome; LM, light microscopy; IF, immunofluorescence; EM, electron microscopy; TBM, tubular basement membrane; EDD, electron-dense deposits; MMF, Mycophenolate mofetil*.

a *Number in parentheses corresponds to reference number*.

The pathogenesis of predominant tubulointerstitial lupus nephritis is still unknown. The deposition of immune complex on TBM may reflect *in situ* formation of immune complexes after the binding of circulating autoantibodies to exogenous or natural antigens since no obvious glomerular lesions were discovered. Recent studies indicated that germinal center-like structure might be formed in the interstitial nephritis of SLE, which could secrete autoantibodies that form immune complex along TBM, activate complement system, and result in focal inflammatory lesions and fibrosis (22). However, this case showed no germinal center-like structure in the kidney biopsies.

## Conclusions

In summary, we reported an old man who presented with acute kidney disease attributable to predominant tubulointerstitial lupus nephritis. Renal biopsy showed almost normal glomeruli but the tubulointerstitium showed “storiform” pattern with interstitial infiltration of IgG3 predominant plasma cells. Immunofluorescence showed IgG and C1q linear and granular staining along TBM and interstitium. He was responsive to medium dose steroids and mycophenolate mofetil. However, the underlying pathogenesis in the predominant tubulointerstitial lupus nephritis still needs to be addressed.

## Data Availability Statement

The raw data supporting the conclusions of this article will be made available by the authors, without undue reservation.

## Ethics Statement

The studies involving human participants were reviewed and approved by Peking University First Hospital, approval number: 2017[1333]. The patients/participants provided their written informed consent to participate in this study. Written informed consent was obtained from the individual(s) for the publication of any potentially identifiable images or data included in this article.

## Author Contributions

YT and YQ analyzed and interpreted the patient data and were major contributors in writing the manuscript. X-jY and S-xW performed interpretation of pathological data. RX, F-dZ, and M-hZ performed interpretation of the clinical data and substantively revised it. All authors contributed to the article and approved the submitted version.

## Conflict of Interest

The authors declare that the research was conducted in the absence of any commercial or financial relationships that could be construed as a potential conflict of interest.

## Supplementary Material

Supplementary Figure 1 **(A)** Immunohistochemical staining of CD3 (×100); **(B)** Immunohistochemical staining of CD20 (×100); **(C)** Immunohistochemical staining of CD138 (×100); **(D)** Immunohistochemical staining of total IgG (×200).
