# Supplementary figures and images for: Case Report: Predominant Tubulointerstitial Lupus Nephritis or the Combination With IgG4-Related Disease?
Source: Front Med (Lausanne). 2021 Jun 28;8:684889. doi: 10.3389/fmed.2021.684889 (PMC8273166; doi:10.3389/fmed.2021.684889)

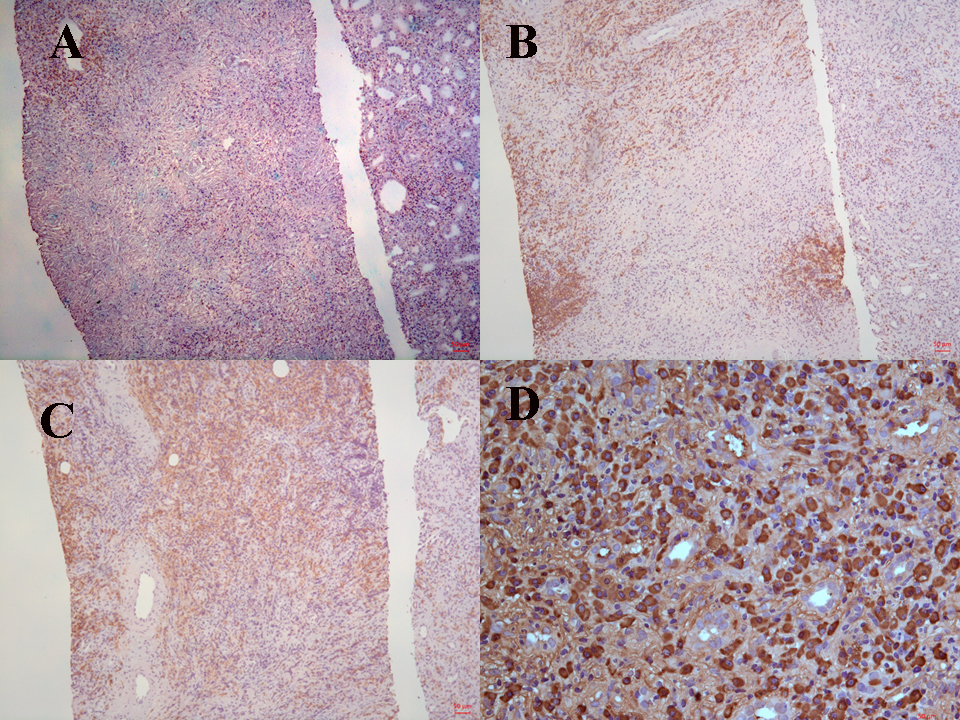

Supplement: Supplementary Figure 1 — (A) Immunohistochemical staining of CD3 (×100); (B) Immunohistochemical staining of CD20 (×100); (C) Immunohistochemical staining of CD138 (×100); (D) Immunohistochemical staining of total IgG (×200). [file Image_1.JPEG]
